# Aldehyde Dehydrogenase 1A1: Friend or Foe to Female Metabolism?

**DOI:** 10.3390/nu6030950

**Published:** 2014-03-03

**Authors:** Jennifer M. Petrosino, David DiSilvestro, Ouliana Ziouzenkova

**Affiliations:** Department of Human Sciences, The Ohio State University, Columbus, OH 43210, USA; E-Mails: petrosino.5@osu.edu (J.M.P.); disilvestro.4@osu.edu (D.D.)

**Keywords:** oestrogen, Raldh, alcohol dehydrogenase, lymphotactin, retinoids, tissue factor, PCOS, gender differences, Esr1, android obesity

## Abstract

In this review, we summarize recent advances in understanding vitamin A-dependent regulation of sex-specific differences in metabolic diseases, inflammation, and certain cancers. We focus on the characterization of the aldehyde dehydrogenase-1 family of enzymes (ALDH1A1, ALDH1A2, ALDH1A3) that catalyze conversion of retinaldehyde to retinoic acid. Additionally, we propose a “horizontal transfer of signaling” from estrogen to retinoids through the action of ALDH1A1. Although estrogen does not directly influence expression of *Aldh1a1*, it has the ability to suppress *Aldh1a2* and *Aldh1a3*, thereby establishing a female-specific mechanism for retinoic acid generation in target tissues. ALDH1A1 regulates adipogenesis, abdominal fat formation, glucose tolerance, and suppression of thermogenesis in adipocytes; in B cells, ALDH1A1 plays a protective role by inducing oncogene suppressors *Rara* and *Pparg*. Considering the conflicting responses of *Aldh1a1* in a multitude of physiological processes, only tissue-specific regulation of *Aldh1a1* can result in therapeutic effects. We have shown through successful implantation of tissue-specific *Aldh1a1*^−/−^ preadipocytes that thermogenesis can be induced in wild-type adipose tissues to resolve diet-induced visceral obesity in females. We will briefly discuss the emerging role of ALDH1A1 in multiple myeloma, the regulation of reproduction, and immune responses, and conclude by discussing the role of ALDH1A1 in future therapeutic applications.

## 1. Introduction

In recent decades, interests have shifted from studies in reproductive biology to a global assessment of sex-specific differences in metabolism and pathogenesis [1]. Numerous studies have demonstrated gender specific differences in susceptibility to immune and metabolic disorders [1,2,3]. Researchers began to recognize that health benefits in men and women were attributed to different types of nutrients. For example, yellow vegetables rich in carotenoids and retinoids are associated with sex-specific patterns of disease prevention [2,4]. Furthermore, yellow vegetables are associated with reduced risk of lung cancer in women [4], whereas in men a diet rich in green xanthophyll carotenoids vegetables does not promote reductions in cardiovascular disease [2]. Thus, there is a vital need to develop prevention and treatment programs dependent upon the mechanisms responsible for divergent metabolic responses between genders. In this review, we discuss the female-specific aspects of vitamin A metabolism and its contributions to the regulation of obesity, immunity, and reproduction.

**Figure 1:**
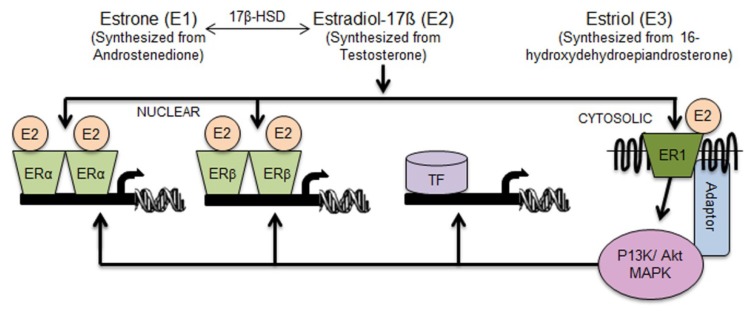
Direct and indirect mechanisms for the regulation of gene expression by estrogen. E1, E2, and E3 are the three predominant naturally occurring forms of estrogen. 17β-HSD catalyzes the reversible conversion of E1 and E2. Three types of estrogen receptors were identified: nuclear receptors ERα and ERβ, as well as transmembrane G-protein coupled estrogen receptor ER1 (alias GPER). The classical pathway for direct gene transcription is the binding of E2 to ERα and ERβ and activation of target genes containing ER response elements (ERE) (Vertical signaling). Activation of ER1 by E2 can in turn activate the classical ERα and ERβ pathways through the signaling cascade governed by PI3K/AKT protein kinases (Vertical signaling). Alternatively, PI3K/AKT triggers activation of other transcription factors (TF) that do not contain ERE (Horizontal signaling). 17β-HSD, 17β-Hydroxysteroid dehydrogenase; ERα, estrogen receptor alpha; ERβ, estrogen receptor beta; GPER, G-protein-coupled estrogen receptor one (alias ER1, GPER or GPR30); P13K, phosphatidylinositide 3-kinase/protein kinase B; AKT, protein kinase B.

The fundamental understanding of sex-divergent responses evolved from major discoveries related to the female sex hormones. This first occurred between 1930 and 1950 when Edward Adelbery Doisy isolated the female sex hormones: estrogen (17-β-estradiol, E2, oestrogen), estron (E1), and estriol (E3) [5]. Years later, nuclear estrogen receptors were cloned and discovered to be responsible for estrogen’s actions [6]. In 1962 estrogen receptor 1 (alpha) (gene nomenclature term *Esr1*, also known as *Era*, *Erα*) was described by Elwood V. Jensen [7]. Following this discovery, in 1996, Dr. Jan-Ake Gustafsson’s laboratory cloned estrogen receptor 2 (beta) (gene nomenclature term *Esr2*, also known as *Erb*, *Erβ*) [8]. E2 is a specific high affinity ligand for these receptors, whereas E3 acts to antagonize E2’s activation of ER [9]. The expression pattern of estrogen receptor *Erα*, and especially *Erβ*, is suggestive of their role in the regulation of metabolic, immune, and reproductive functions [6] (Figure 1). The genome-wide location of transcription factor binding-sites or histone modifications (cistrome) have proven that ERα responses are cell-type specific in tissue. This is the result of ERα localization in response to different epigenetically marked cis-regulatory regions of target genes [10]. Furthermore, suppression of genes in these regions by ERα regulates tissue-specific responses to E2 that are dependent on concurrent cooperation among transcription factors and coactivators [11,12]. Together, these E2-regulated events contribute to tissue-specific responses.

The dependence of metabolic [13,14] and inflammatory [15] processes on ERs in both genders was later confirmed in mouse models deficient in *Erα*, *Erβ*, or both [16]. The absence of *Erα* in both male and female mice resulted in increased white adipose tissue mass (WAT) and impaired insulin resistance [16,17,18]. In ovariectomized mice, both E2 and *Erα* are necessary to overcome insulin resistance induced by a high-fat diet [19]. In contrast, *Erβ*^−/−^ mice are resistant to age-induced obesity ([16], reviewed in [6]), suggesting differences between the metabolic roles of ERα and ERβ. In addition, E2 can induce potent anti-inflammatory and neuroprotective effects via ERα-mediated mechanisms in female mice with pronounced autoimmune encephalomyelitis [20,21]. Previous experiments examining E2 in *Erα*^−/−^ and *Erβ*^−/−^ mice showed a spectrum of responses that occur as a result of both E2-dependent and ER-independent mechanisms. Furthermore, later investigation revealed that E2 can also operate through a transmembrane G protein-coupled receptor 1 (ER1, GPER; formerly known as GPR30) to induce rapid signaling events [22] (Figure 1). The ER1-mediated phosphorylation cascade leads to activation of the nuclear ERα and ERβ (vertical signal transfer) and other families of transcription factors (horizontal signal transfer).

To understand sex-specific tissue effects, varying susceptibility to diseases, and distinctive responses to nutrients in males and females, other genomic and non-genomic mechanisms of E2 action must be explored. In this review, we discuss the relationship between E2 and vitamin A pathways in the context of our recent work investigating female-specific mechanisms for: “thrifty” metabolism; central-to-peripheral fat distribution; cytokine production; and immune responses [23,24,25,26,27].

We propose a novel mechanism for horizontal auto-/paracrine signal transfer from E2 to retinoic acid (RA) and RA-dependent transcriptional network that regulates tissue responses in a sex- and tissue-specific fashion.

## 2. Review Section

### 2.1. Aldh1 Family of Enzymes: Function and Regulation by E2

Retinoic acid (RA) production begins with the conversion of the vitamin A metabolite, retinol (ROL), to retinaldehyde (Rald) by the alcohol dehydrogenase (ADH) family of enzymes (reviewed in [28]). Several microsomal enzymes of the short chain dehydrogenase (SDR/RDH) family convert ROL to Rald *in vitro* [28,29] This conversion of retinol to Rald is a reversible step, carried out by ADHs, RDHs, DHRS9, and some aldo-keto reductases like AKR1B1 [30,31]*.* The cytosolic aldehyde dehydrogenase 1 enzyme family (ALDH1, alias RALDH) catalyzes the final irreversible step in RA production by oxidation of Rald to RA [28,32]. These enzymes in the ALDH1 family possess the same biochemical functions (retinoic acid production), but display different physiological functions [28,32]. Dissimilar functions of ALDH1 enzymes in adipose tissue will be discussed in the following section.

**Figure 2:**
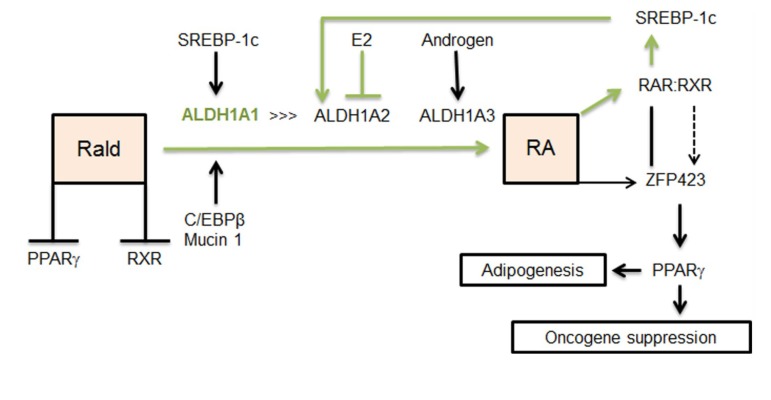
Sex hormones and SREBP selectively regulate expression of *Aldh1.* ALDH1A1, ALDH1A2, and ALDH1A3 are members of the ALDH1 enzyme family that convert Rald to RA. E2 suppresses *Aldh1a2* and *Aldh1a3* in adipose tissue. Androgens can stimulate *Aldh1a3* expression. *Aldh1a3* expression is elevated in the visceral fat of ovariectomized mice. Hypothetical feed-forward pathway for *Aldh1a1* regulation in females (*green line*). SREBP-1c can up-regulate *Aldh1a1* and *Aldh1a2* in animals fed a high-fat diet. However, in females E2 can suppress *Aldh1a2*. RA produced by ALDH1A1 can activate the RAR/RXR transcriptional complex, which in turn activates SREBP-1c. C/EBPβ and mucin1 can regulate *Aldh1a1* in breast cancer cells. RA production can stimulate adipogenesis through ZFP423 and PPARγ. In multiple myeloma B cell lines, RA suppresses oncogenes (*Hoxa10* and *Ap1*) through ZFP423 and PPARγ activation. Rald suppresses adipogenesis through inhibition of RXR and PPARγ. Rald, retinaldehyde; ALDH1A1, retinaldehyde dehydrogenase 1 family, member A1; ALDH1A2, retinaldehyde dehydrogenase 1 family, member A2; ALDH1A3, retinaldehyde dehydrogenase 1 family, member A3; SREBP-1c, sterol regulatory binding protein 1; C/EBPβ, CCAAT/enhancer-binding protein beta; RA, retinoic acid; ZFP423, zinc finger protein 423; PPARγ, peroxisome proliferator-activated receptor gamma; RXR, retinoid X receptor.

RA induces differentiation of cells and regulates gene transcription, signaling events, and post-translational modification of proteins (reviewed in [26,33]). All isomers of RA are high affinity ligands for the retinoic acid receptor (RAR) (reviewed in [34]). RAR can heterodimerize with retinoic X receptor (RXR), another nuclear receptor that binds to 9-*cis* RA. This heterodimer then binds to RA response elements (RARE) and regulates gene expression [35]. Furthermore, RA regulates expression of peroxisome proliferator–activated receptor γ (*Pparg*) [29], the master regulator of adipogenesis, via activation of transcription factor *Zfp423* [24,36] (Figure 2). In contrast, Rald suppresses adipogenesis by inhibiting PPARγ and RXR activation [37]. PPARγ, RAR, and RXR regulate numerous differentiation processes and immune response [26,33,34,35]. Considering the role of ALDH1in controlling RA and Rald concentrations, it is possible that ALDH1 plays a role in the auto-/paracrine switch regulating adipogenesis and various other differentiation processes. RA production must be regulated in a spatiotemporal fashion due to the abundant yet versatile physiological activity of RA [38]. The ALDH1 family is comprised of three enzymes: ALDH1A1, ALDH1A2, ALDH1A3 [32,38]. All the three isoforms exist in mice, chickens, and humans and their sequences are conserved between species [38]. Murine ALDH1A1 is a 511 amino acid protein that is 87% identical to human ALDH1A1, murine ALDH1A2 is a 518 amino acid protein that is 97% identical to its human counterpart, and lastly, murine ALDH1A3 contains 512 amino acids and is 94% identical to the human protein (NCBI/BLAST database). ALDH1 enzymes share similar properties, such as conversion of Rald to RA but have unique biochemical and physiological functions (reviewed in [38]).

**Figure 3:**
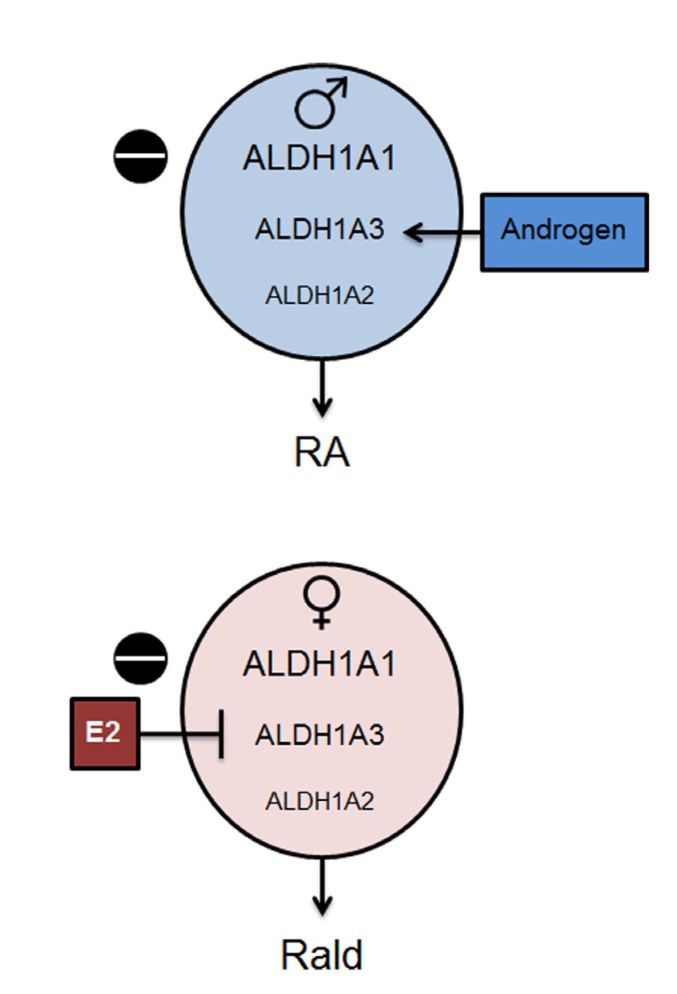
Schematic presentation of the sex-specific change in the retinoid balance in male and female VF. In VF of mice, *Aldh1a1* was predominantly expressed compared to *Aldh1a2* and *Aldh1a3* (indicated by size of the letters). *Aldh1a2* and *Aldh1a3* levels are lower in females (pink circle) compared to those seen in males (blue circle) due to suppression of *Aldh1a3* by estrogen. Deficiency in *Aldh1a1* (*black circle with “−” symbol*) further reduces expression of *Aldh1a2 and Aldh1a3* in females, which leads to decreased Rald catabolism. Rald was detected in the VF of female mice. In *Aldh1a1*^−/−^ male mice, *Aldh1a3* expression is under androgen control; it can catalyze RA production from Rald in *Aldh1a1*^−/−^ males.

All ALDH1 enzymes catalyze the formation of Rald to RA [38]. Hierarchy of *Aldh1* activation, their expression levels in different tissues, and contribution to RA production is regulated by sex hormones and nutrients, such as cholesterol and fatty acids [27,39,40,41]. Estrogen response element sites exist in the promoter of *Aldh1a2* [39], while *Aldh1a3* is regulated by androgens via androgen receptors [41] (Figure 3). In our studies, ovariectomy enhanced *Aldh1a3* expression in retroperitoneal adipose tissue; while both *Aldh1a2* and *Aldh1a3* were expressed at lower levels than *Aldh1a1* in a control female group [27]. The E2-dependent regulation of *Aldh1* occurs in a tissue specific manner. For example, in the uterus of ovariectomized rats, E2 stimulation markedly increases *Aldh1a2* expression but suppresses *Aldh1a1* expression [42]. The dissimilar response of tissues to E2 depends on the presence of transcription factors regulating *Aldh1*. In breast cancer cells, the *Aldh1a1* gene promoter is activated by a transcriptional complex of phosphorylated CCAAT/enhancer-binding protein β (C/EBPβ) and mucin 1 [43]. In addition, the promoter of *Aldh1a1* and *Aldh1a2* can be activated by sterol regulatory element binding protein 1 (SREBP-1c; nomenclature SREBF1) and to a lesser extent by SREBP2 that binds to SREBP regulatory element sites (SRE) [27,40] (Figure 2). The up-regulation of RAsynthesis [27] and *Aldh1* expression in response to high-fat and high-cholesterol diets [40] can involve SREBP. In turn, RA, Rald, and ROL regulate the expression of *Srebp-1c* [44] through RXR [45], and possibly, RAR dependent mechanisms. This suggests feed-forward regulation of *Aldh1a1* and *Aldh1a2* through SREBP-1c. In consonance with this hypothesis, *Aldh1a1* deficiency in mice reduces RA production and the expression of *Aldh1a2* and *Aldh1a3* [27,46]. Examination of the relative contribution of all transcriptional pathways to regulation of *Aldh1* expression could provide further insight about *Aldh1* regulation by nutrients in males and females.

Although the gene members of the *Aldh1* family are expressed quantitatively in a tissue-specific manner [38], in the majority of adult tissues, all three isoforms of *Aldh1* are expressed. Specifically, *Aldh1a1* is ubiquitously expressed at higher levels than *Aldh1a2* and *Aldh1a3* in all tissues except the arachnoid membrane in the brain and some other cell types (Ziouzenkova, unpublished observations). The functional importance of expression of the three *Aldh1* family members at different levels is poorly understood, and thought to be reserved for the compensatory RA production. In males, RA production is dependent on both *Aldh1a1* and *Aldh1a3*. However, in females, *Aldh1a1* is the predominant enzyme for RA production [24,27], because both *Aldh1a2* and *Aldh1a3* are suppressed by E2 [27] (Figure 3). *Aldh1a1* becomes a critical downstream pathway mediating metabolic sex-specific differences since obesogenic and inflammatory responses to a high-fat diet are averted in *Aldh1a1*^−/−^ females but not in *Aldh1a1*^−/−^ males [27]. Based on our studies in adipose tissues, we propose a sex hormone-dependent “exclusion model” for the regulation of the ALDH1 family of enzymes that alter Rald and RA concentrations in a sex-specific manner (Figure 3). We expect this pattern to be present in tissues in which expression of nuclear and cytosolic ER and other transcription factors enables expression of only one *Aldh1* enzyme. The predominant expression of *Aldh1a1* in female adipose tissue [27] offers an opportunity to test the contributions of the vitamin A pathway to the regulation of female-specific responses in VF.

### 2.2. Aldh1 Regulation of Female-Specific Fat Distribution

Women tend to have higher fat mass than men throughout their entire lifespan [47,48]. Statistically, there are higher rates of obesity in women than men (61.3% *vs.* 42%). Obesity in women is correlated with an increased risk of diabetes mellitus, cardiovascular disease, cancer, and premature death [49,50,51]. The differences in metabolic rate [52,53,54] and energy homeostasis between males and females is dependent on actions of sex hormones [55,56,57] and expression of ER receptors in different tissues, including muscle [56,58] and adipose [59,60,61,62,63,64,65]. While the influence of ALDH1 enzymes in muscle remains unclear, current research has demonstrated that they have a critical regulatory role in adipose tissue [24,27].

Fatty Acids (FA) are stored as triglycerides in humans in three types of WAT: subcutaneous (SF), omental, and mesenteric. Given the central location of omental fat around viscera, this fat depot is also referred to as visceral (VF), central, abdominal, and trunk fat. Based on both heritable developmental gene expression and their striking physiological differences, these fat depots are proposed to be differentiated as separate tissues [66]. Additionally, WAT has a rare population of thermogenic cells known as thermocytes, bright, or beige cells [67]. Thermocytes, which directly contribute to basal energy expenditure, can reduce propensity for obesity [67].

It has been well established that fat distribution in the abdominal visceral region is typically attributed to males and it is often termed “android obesity” [60]. Young healthy females usually exhibit a different fat distribution pattern, which is characterized by increased SF relative to VF, especially in the lower body [48,68,69,70,71,72]. This pattern of fat distribution is known as “gynoid obesity”. On a regular diet, WAT is predominantly formed in SF depot in females and VF depot in males [73]. In women with polycystic ovary syndrome (PCOS), excessive androgen production leads to the development of “android” visceral obesity [48,74,75,76,77]. However, development of android obesity in women can be reverted through administration of E2 [48,78,79,80,81,82], whereas androgen replacement is associated with increased VF formation in these patients [83]. The sulfoconjugation of E2 in female mice overexpressing estrogen sulfotransferase does not influence VF; however, it does decrease SF depots by suppressing adipogenesis and impairing insulin sensitivity [84]. Physiologically, the postmenopausal period is marked by E2 deprivation and a significant increase in VF accumulation [4,85]. In experimental rodent studies, ovariectomy is the most commonly used method to study the effects of E2 deprivation [4]. We have found that ovariectomy is associated with increased expression of *Aldh1a3* in the VF, although *Aldh1a1* remained the most predominantly expressed member of the ALDH1 family. Deficiency in *Aldh1a1* effectively protects female mice against VF formation induced by ovariectomy [27]. In healthy women, *Aldh1a1* mRNA expression is the most abundant compared to other *Aldh1* genes, in both VF and SF [27]. Considering this, it appears that *Aldh1a1* is a central downstream pathway through which E2 regulates VF formation in female mice and possibly postmenopausal women.

### 2.3. Aldh1 Role in Sex-Specific Diet-Induced Thermogenic Regulation

Diet modifies sex-specific fat distribution [86]. On a high-fat diet, females exceedingly accumulate VF, which increases their risks for type 2 diabetes and cardiovascular disease. Imbalance of energy storage and expenditure is the accepted cause of obesity [87]. This is commonly interpreted as increased food intake *vs.* decreased physical activity. However, this imbalance in energy storage and expenditure can also affect regulatory mechanisms in adipose tissue leading to suppression of thermogenic energy expenditure and activation of lipogenic and lipid storage processes [87]. In females, this type of deregulation contributes to the decrease in basal metabolic rate and increase in visceral obesity on high-fat and Western diets [52]. ERβ is involved in the regulation of metabolic rate and can suppress diet- and ovariectomy-induced obesity in mice [88]. In obese ovariectomized mice, E2 effects depend on *Ppara* [89]. Downstream mechanisms by which ERα regulates these processes are also poorly understood and may involve regulation of the transcriptional co-activator of PPARγ, PGC-1α (nomenclature PPARGC1) [90]. Our recent studies [27] have demonstrated that ALDH1 enzymes can act downstream of sex-hormone responses and switch the lipogenic processes to thermogenic.

*In vitro*, *Aldh1a1* expression is increased during adipocyte differentiation and contributes to ~70% RARE activation [24]. Knocking out *Aldh1a1* in mice demonstrates how prevention of lipogenic adipogenesis in adipocyte cultures results in the acquisition of thermogenic characteristics [25]. Additionally, differentiated *Aldh1a1*^−/−^ cells compared to WT or 3T3-L1 adipocytes display higher expression of *Ucp1*, *Pgc-1α*, and *CoxIV* as well as down-regulation of lipogenic genes *Pparg*, *Fas* and *Fabp4* [24,25]. These results are in agreement with findings *in vivo*, where thermogenic remodeling occurs in the VF of *Aldh1a1*^−/−^ females [27]. Thermogenesis does not occur in the VF of *Aldh1a1*^−/−^ males expressing the two other RA producing enzymes, *Aldh1a2* and *Aldh1a3*. This sex-specific phenomenon results in greater weight loss in the VF depots of *Aldh1a1*^−/−^ females compared to their male counterparts [27]. RA production and effects are also depot-specific in females. In females, RA is produced in VF primarily through the action of *Aldh1a1*, whereas RA in SF is produced by *Aldh1a1* and *Aldh1a3* [24,27]. Due to depot-specific expression of *Aldh1* enzymes, *Aldh1a1* deficiency influences formation of the VF depot more than SF [24,26,27]. Moderate supplementation with vitamin A does not result in alterations to this fat distribution, even when concentration is increased up to 5-fold [23]. However, the implantation of *Aldh1a1*^−/−^ cells into the VF of WT female mice induces similar thermogenic modification of adipocytes as seen in whole body *Aldh1a1* deficient female mice [25]. This increased thermogenesis in VF results in the loss of VF mass in treated females despite consumption of a high-fat diet [25]. Thus, the lack of a single *Aldh1a1* gene in a minor population of adipocytes is capable of abrogating the “thrifty” female metabolism via increased thermogenesis in VF.

The mechanism of this profound thermogenic remodeling of adipose tissue in *Aldh1a1*^−/−^ females appears to stem from autocrine displacement of RA with Rald (Figure 3). In *Aldh1a1*^−/−^ females, deficient expression of all ALDH1 enzymes abolishes Rald catabolism and RA production [37]. As a result, this modified signaling landscape leads to dysregulation of both genomic and non-genomic events. In *Aldh1* deficient female adipocytes, Rald rapidly (within 30 min) increases translation of ATGL protein and induces lipolysis [27]. ATGL-mediated hydrolysis of fatty acids activates PPARα and expression of its target genes, *Ucp1*, *Pgc-1α*, and *CoxVI* [91,92]. In *Aldh1a1^−/−^* male adipose tissue explants, stimulation with Rald can recapitulate increase in ATGL-mediated lipolysis [27]. Thus, non-genomic effects on lipolysis appear to be dependent on the presence of Rald. Genomic *Aldh1* effects in adipose tissue depend on: (1) RA regulating expression of *Zfp423* and *Pparg* [24]; and (2) Rald inhibiting PPARγ and RXR activation [37]. An alternative explanation for thermogenic mechanisms in *Aldh1a1^−/−^* females, as proposed by Kiefer *et al.* [93], would be through the binding of Rald to RAR. However, Rald is known as a weak agonist for the RAR [94] and is inferior to RA in mediating RAR-dependent transcription. RA is abundantly produced by all three ALDH1 enzymes in WT, but is not produced in *Aldh1a1*^−/−^ VF. In addition, it is unclear how this proposed Rald/RAR receptor interaction could explain increases in ATGL protein levels in the presence of cycloheximide that occur without changes in mRNA expression [27]. RA can activate RAR and induce thermogenesis in animals treated with high concentrations of RA [95].

Humans, similar to mice, exhibit enhanced lipolysis in the presence of Rald than RA *in vitro* [27]. Stromal vascular cells isolated from obese women, but not men, had significantly greater expression of *Aldh1a1* suggesting that ALDH1A1 function in the regulation of adipocyte biology is conserved between species [27]. It seems plausible that *Aldh1a1* deficiency in human omental fat or implantation of *Aldh1a1*^−/−^ deficient pre-adipocytes (as in [25]) could serve as a potential sex-specific therapeutic strategy to combat android obesity in women. Cells encapsulated in alginate poly-l-lysine have been tested and implanted and used for long-term (9.5 years) xenotransplantation of pancreatic β-cells producing insulin in human patients [96]. The successful application of encapsulated *Aldh1a1*^−/−^ cells for induction of thermogenesis in mice then raises the question: can these implants be similarly effective for the reduction of deleterious VF in women?

### 2.4. Aldh1 Sexual Dimorphism of WAT and Risks for Chronic Diseases

Fat depot distribution is an important risk factor for chronic metabolic diseases and particular cancers. Research has shown that VF (omental in humans and retroperitoneal in mice) is associated with agreater risk of developing metabolic syndrome [48,70,97]. VF and SF produce dissimilar levels of signaling molecules, including hormones and adipokines, that can further modify fat distribution patterns [98,99,100]. Thus, sex-specific fat distribution and adipose tissue mass in each depot contribute to sexual dimorphism of adipokines in blood [48]. The retinoid-relevant cytokines include retinol binding protein 4 (RBP4), dipeptidyl-peptidase 4 (DPP4), chemerin (formerly known as tazarotene-induced gene 2 protein (TIG2) and retinoic acid receptor responder protein 2 (RARRES2)), and lipocalin 2 (LCN2) [48]. Cytokines from VF act in a pro-inflammatory manner and contribute to insulin resistance. Females tend to have higher SF mass and insulin sensitivity [97,101] possibly due to the adiponectin production in SF [102,103]. Recently, many comprehensive reviews have discussed the collective influence of sex hormones and retinoids in the regulation of cytokine production and secretion in various tissues, including adipose (reviewed in [97,104,105,106]). The extent of ALDH1A1’s influence can be better explained through the differences in plasma cytokine profiles of WT and *Aldh1a1*^−/−^ mice on a high-fat diet [23].

It appears that *Aldh1a1* contributes to a sex-specific adipokine/cytokine profile in plasma by mechanisms dependent and independent of adipose tissue remodeling. The observed reduction in levels of SF and VF in *Aldh1a1*^−/−^ compared to obese WT mice were associated with reduced adipokine secretion of adiponectin, leptin, and various pro-inflammatory cytokines including monocyte chemoattractant protein-1 (MCP1) and chemokine (C–X–C motif) ligand 10 (CXCL10; also known as IP-10). However, these changes were similar between genders and showed a positive correlation between MCP1 and IP10 cytokines and adipose tissue mass [23]. In contrast, tissue factor (TF) and factor VII reduction was specific to the *Aldh1a1*^−/−^ female group**, **and did not correlate with adipose tissue mass [23]. The lack of correlation between these factors and adipose tissue mass suggest participation of other tissues. Therefore, in other tissues ALDH1A1 can regulate production or secretion of coagulation proteins in female mice and possibly control thrombogenic events. The relevance of these observations for wound healing or cardiovascular events requires further examination to understand the mechanism by which ALDH1A1 regulates TF and factor VII. For example, *Aldh1a1* is expressed and responsible for the high-fat diet-induced steatosis in the liver [37]. TF and factor VII synthesis occurs in hepatic cells. This link between ALDH1 enzymes and acute response proteins could elucidate the role of *Aldh1a1* in liver disease.

The *Aldh1a1*-dependent regulation of cytokines also contributes to improved glucose sensitivity in *Aldh1a1*^−/−^ on a high-fat diet [23]. Previous research has suggested that there is a pivotal hepatic role in establishing insulin sensitivity in *Aldh1a1*^−/−^ mice [107]. However, our recent studies have shown that engrafted *Aldh1a1*^−/−^ cells in VF also improve glucose tolerance in obese WT females [25]. This finding, in line with numerous others, implicates VF in the development of insulin resistance [4,82]. Paradoxically, HOMA index assessments revealed that *Aldh1a1*^−/−^ males exhibit more pronounced improvement of glucose metabolism than *Aldh1a1*^−/−^ females, as a result of lower plasma insulin levels observed in *Aldh1a1*^−/−^ females compared to *Aldh1a1*^−/−^ males [23]. Recent studies have found 9-*cis* RA in insulin-producing pancreatic β-cells [108]. In light of these findings, the influence of RA and ALDH1 enzymes over insulin production and secretion in males and females needs to be examined. Although more work needs to be performed in the future, the central role of ALDH1 in sex-specific auto-/paracrine control of obesity, glucose metabolism, thrombogenic factors, and inflammation is evident.

### 2.5. Aldh1 Control of Sex-Specific Reproduction

Meiosis is a key process in mammalian reproduction that leads to formation of spermatogoniam and oocgonia. Meiosis signifies the first sex-specific differentiation of primordial germ cells (PGC). PGC’s development into gametes at the completion of meiosis is dependent on sex-chromosome composition [109,110,111,112]. Additionally, the time sensitive induction of PGC sexual differentiation is influenced by the environment and localization of cells in gonads [113]. Meiosis occurs prenatally in females and postnatally in males. Early studies have shown that regardless of XX or XY chromosomal composition in PGC, their localization in the ovary, but not in the testes can initiate meiosis [114,115]. Thus, the environment in the gonad (mesonephroi) plays a regulatory role and produces paracrine factors that influence PCG initiation into meiosis. However, the question remains: why does meiosis occur prenatally in females, but postnatally in males?

PGC contains meiosis commitment genes, including *Stra8* in vertebrates [116]. *Stra8* is expressed in both the cytoplasm and nucleus [117]. In response to RA, STRA8 initiates meiosis in stimulated male and female PGC cells *in vitro* [116,118]. *In vivo*, *Stra8* mRNA expression coincides with the onset of meiosis in postnatal testes and prenatal ovaries [118]. The specific mechanism by which STRA8 regulates meiosis has not yet been identified. *Stra8* regulates chromosomal condensation and formation of synaptonemal complexes. A search for an enzymatic switch releasing RA to initiate meiosis led to the identification of ALDH1A2 (RALDH2) as the principal enzyme regulating RA synthesis in the gonadal mesonephroi [119,120,121,122]. Studies conducted with *Aldh1a2* and *Aldh1a3* double knockout mice crossed with RARE reporter mice have revealed that meiosis in females can occur in the absence of detectable RA production by these enzymes. In postnatal testes, the critical role of RA for meiosis has been supported by numerous genetic and pharmacological studies. In male mesonephros, ALDH1A2 governs RA production. Expression of *Cyp26B* in prenatal PGCs catabolizes RA. In the postnatal period, paracrine RA signals are required for meiosis. The unexpected lack of RA during meiosis in females leaves unanswered questions about alternative paracrine initiating factors (reviewed in [119]) and/or alternative vitamin A metabolites activating *Stra8* in the ovary.

The analytical sensitivity for the detection of RA production in mesonephroi for PGC activation can be a technical issue responsible for the paradoxical finding in *Aldh1a2* and *Aldh1a3* double knockouts. However, the indirect role of RA in female meiosis is supported by a robust inhibition of meiosis in fetal ovaries by an antagonist of RAR [123,124]. An important shortcoming of previous genetic studies was the low expression levels of *Aldh1a1* enzymes producing nanomolar concentrations of RA. At these levels, RA is not detectable in RARE reporter models, but capable of meiotic induction. Meiosis in the human ovary is accompanied by increased *Aldh1a1* expression in agreement with a suggested requirement of RA synthesis for meiosis [125]. Recently, *Aldh1a1* has been shown to have sex specific influences on female biology [51], possibly warranting further investigation into its contribution to female reproduction.

## 3. Conclusions

**Figure 4:**
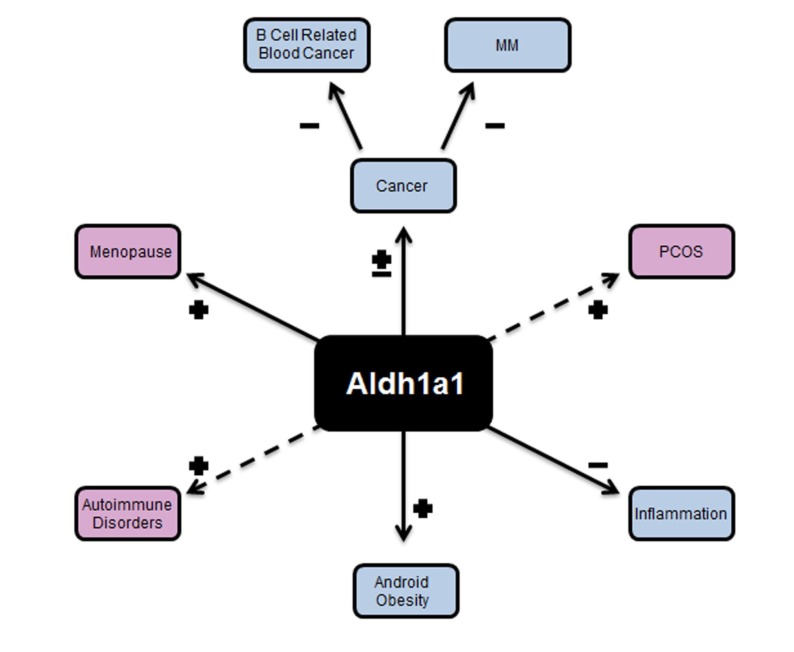
The spectrum of disorders that are influenced by ALDH1A1. Android obesity, which is marked by the accumulation of fat around the inner organs (viscera) is dependent on *Aldh1a1*. In the figure, (+) represents upregulation of the gene in a specific disease, where as (−) represents down-regulation of this gene and elucidates that up-regulation or down-regulation of the gene contributes to development of the disease or condition. Visceral fat is decreased in *Aldh1a1*^−/−^ female mice and also in obese WT female mice treated with *Aldh1a1*^−/−^ preadipocytes. Visceral obesity is developed in the absence of estrogen in postmenopausal women and in patients with PCOS. Inflammation and B cell differentiation is regulated by RA, and depends on ALDH1A1. RA regulates tumorigenesis. *Aldh1a1* expression is markedly reduced ((−) in the figure) in multiple myeloma (MM) and other blood cancers. In contrast, breast cancer cells have elevated *Aldh1a1* expression.

(1) Sex-hormones regulate a tissue-specific pattern of Aldh1 enzyme expression.

(2) ALDH1A1 is the predominant enzyme for the regulation of RA and Rald concentrations in female tissues.

(3) The regulation of sex differences could be mediated by E2 and/or be transferred to retinoid-dependent pathways.

(4) Aldh1a1 deficiency in female adipose tissue improves cytokine profile and metabolic disorders such as visceral obesity and glucose tolerance (Figure 4).

The genetic deficiency in *Aldh1a1* may have potential therapeutic applications in adipose tissue [25]. However, physiological levels of body fat need to be maintained to avoid insulin resistance, leptin deficiency and dyslipidemia that is associated with lipodistrophy [126]. However, the disruption of *Aldh1a1* signaling in other tissues can have deleterious effects. Our recent studies showed that deficiency in *Aldh1a1*, but not in *Aldh1a2* and *Aldh1a3*, is the characteristic feature of B cell cancers in humans, especially multiple myeloma (MM) [127]. In B cells, the lack of *Aldh1a1* results in decreased *Rara* and *Pparg* expression that leads to the up-regulation of oncogenes *Hoxa10* and *Ap1* [127].

RA regulates a network of transcription factors and is a powerful therapeutic agent [33]. However, administration of RA is unspecific and causes numerous side effects known as “RA syndrome”. The targeted regulation of ALDH1 enzymes in females or implantation of *Aldh1a1*^−/−^ cells [25] could create an opportunity to regulate RA synthesis in a physiological tissue-specific manner for therapeutic purposes.

### Perspectives

PCOS affects approximately 6.5% of reproductive age women [128,129] and results in development of secondary male sex characteristics, including hyperandrogenism and anovulation. This often leads to amenorrhea, oligomenorrhea, dysfunctional uterine bleeding, and infertility [130]. In addition, PCOS shares many common traits of metabolic syndrome including: visceral obesity, insulin resistance, hyperinsulemia, greaterrisk of diabetes mellitus, and cardiovascular disease [131,132]. Obesity in PCOS patients can present in adolescence prior to menarche [130] suggesting that PCOS dysregulation occurred long before puberty.

Postmenopausal obesity is also a predictor of poor survival rates in women with breast cancer [133]. A strong association was found between family history of breast cancer and a high frequency of *Aldh1a1* expression in breast ductules in women, regardless of a hereditary breast-ovarian cancer syndrome status (*Brca1* and *Brca2* mutations), age, parity, or occurrence of cancer [134]. The role of *Aldh1a1* in these diseases warrants investigation, considering the central role of *Aldh1a1* in ovariectomy-induced visceral obesity [27]. We have explored the therapeutic application of *Aldh1a1*^−/−^ preadipocytes and observed delayed onset of visceral obesity in WT female mice after implantation of biocompatible encapsulated *Aldh1a1*^−/−^ cell [25]. Potentially, similar therapies could be developed for PCOS patients.

**Figure 5:**
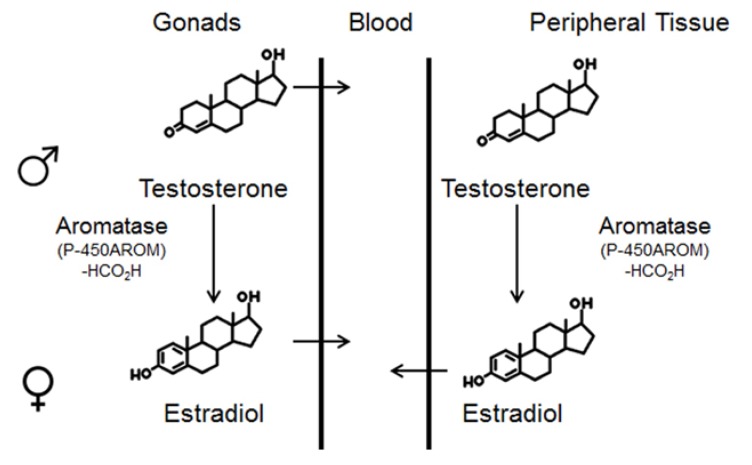
Schematic presentation of sex hormone production in gonads and peripheral tissues. Sex hormones are secreted from the male and female gonads. The testes secrete testosterone into the blood stream while in the ovaries aromatase converts testosterone to estradiol prior to secretion into the blood stream. In the peripheral adipose tissue, aromatase converts testosterone to estradiol.

PCOS manifestation often includes development of hypertrophied ovarian theca cells [135] that express steroidogenic enzymes for androgen biosynthesis: CYP11A1, CYP17, HSD3B2 [136] 5a-reductase, and 5a-androstane-3,17-dione [135,137]. Additionally, 5a-reductase increases in PCOS granulosa cells leads to elevated 5a-androstane-3,17 dione concentrations which then inhibit aromatase and reduces estrogen production in granulosa cells (Figure 5) [137]. Hyperandrogenism in PCOS may also be a function of increased production of biologically active retinoids by theca cells [135]. PCOS theca cells express a set of vitamin A enzymes that are sufficient for atRA biosynthesis, including retinol dehydrogenase 2 (*Rdh2*), retinol dehydrogenase (*RoDH4,2*), cellular retinoic acid binding protein II (*Crabp II*), and prostate short chain dehydrogenase/reductase (*Psdr2*) [138], and *Aldh1a3* [128]. However, retinol is converted to retinaldehyde at an increased rate in PCOS *vs.* normal theca cells [138], suggesting enzymatic dysregulation. In these cells, atRA increases expression of 17 alpha-hydroxylase and androgen production via GATA6 dependent mechanisms [138]. RA also induces expressions of *CYP17* and *CYP11A1* that produce DHEA [139]. Interestingly, in PCOS theca cells, promoters of steroidogenic enzyme expression are up-regulated by retinol, while in healthy women these cells responded only to RA [135]. Thus, ALDH enzymes may have a major role in the imbalance of retinoid concentrations that induce androgen production and steroidogenic enzyme expression in theca cells. Many factors, including production of luteinizing hormones, seem to be activating, mediating and facilitating PCOS, yet RA remains to be a co-regulator of all these processes. Thus, the role of endogenous retinoids in these processes needs to be examined as a potential candidate pathway regulating hormonal balance. A critical understudied aspect of ALDH1A1 function includes its possible catabolic role in different other pathways. While ALDH1A2 and ALDH1A3 specifically utilize Rald as a substrate [32,140], ALDH1A1 utilizes Rald as a preferred substrate at nanomolar concentrations. However, ALDH1A1 also utilizes other aldehyde substrates if they are present at micromolar concentrations *in vitro* [46,141]. *In vivo*, *Aldh1a1*^−/−^ created reductions of physiological levels of retinoic acid and 3-deoxyglucosone [46,142], but it remains unclear whether this metabolic product of glucose oxidation contributes to the metabolic phenotype observed in *Aldh1a1*^−/−^ mice. In adipocyte cultures, 70% of the RA-dependent RARE activation depends on *Aldh1a1* expression. Thus, it appears that ALDH1A1 is a major enzyme contributing to endogenous RA production in adipogenesis in females. However, the environmental condition increasing concentrations of other aldehydes can potentially disrupt RA production in females and increase their susceptibility to autoimmune disorders and blood cancers (Figure 4).

## Abbreviations

3β-HSD: 3β-Hydroxysteroid dehydrogenase type II enzymes
ADH: Alcohol dehydrogenase
Adh1: Alcohol dehydrogenase 1
*Aldh1a1*^−/−^: Knockout in *Aldh1a1*
Aldh1a1: Aldehyde dehydrogenase 1 family, member a1
Aldh1a2: Aldehyde dehydrogenase 1 family, member a2
Aldh1a3: Aldehyde dehydrogenase 1 family, member a3
AP-1: Activator protein 1
ATGL: Adipose triglyceride lipase
BAT: Brown adipose tissue
BMI: Body mass index
COX2: Prostaglandin-endoperoxide synthase 2
CRABP: Cellular retinoic acid binding protein
CYP17: Cytochrome P450 17
CYP1711A1: Cytochrome P450 family 17 subfamily A polypeptide 11
CYP26B1: Cytochrome P450 26B1
CYP450: Cytochrome P450
CXC: CXCL Chemokine group of cytokines
C/EBPβ: CCAAT/enhancer-binding Protein β
DHEA: Dehydroepiandrosterone
DPP4: Dipeptidyl peptidase-4 also known as CD26
E1: Estron
E2: Estradiol
E3: Estriol
ERα: Estrogen receptor alpha
ERβ: Estrogen receptor beta
*Erα*^−/−^: Knockout in *Erα*
*Erβ*^−/−^: Knockout in *Erβ*
ERE: Estrogen response element
FABP: Fatty acid binding protein
FSH: Follicle stimulating hormone
GATA: GATA-binding factor 1
GPER: G protein-coupled estrogen receptor 1
HSD3B2: 3beta-hydroxysteroid dehydrogenase-delta 5-delta 4 isomerase type II
IL-6: Interleukin-6
IFNγ(IFNg): Interferon gamma
IP-10: Inducible Protein-10
LH: Luteinizing hormone
LPS: Lipopolysaccharide
MCP-1: Monocyte chemoattractant protein-1
NF-κB: Nuclear factor kappa-light-chain-enhancer of activated B cells
PCOS: Polycystic ovary syndrome
PGC: Primordial germ cell
PGC-1A (PPARGC1A): Peroxisome proliferator-activated receptor, gamma, Coactivator 1 alpha
PPARα: Peroxisome proliferator-activated receptor alpha
PPARγ: Peroxisome proliferator-activated receptor gamma, *Pparg*
RAR: Retinoic acid receptor, *Rara*
RARE: Retinoic acid response elements
Rald: Retinaldehyde, retinal
RA: Retinoic acid
RoDH: Retinol dehydrogenase
ROL: Retinol
RXR: Retinoid X receptor
SF: Subcutaneous fat
SREBP-1c (SREBF1): Sterol regulatory element-binding protein isoform 1 C
STRA8: Stimulated by retinoic acid 8
STAR: Steroidogenic acute regulatory protein
STAT: Signal transducer and activator of transcription
TF: Tissue factor
UCP1: Uncoupling protein 1
VF: Visceral fat (in this paper mouse retroperitoneal and human omental)
WAT: White adipose tissue
ZFP423: Zinc-finger transcription factor 423
5a-r: 5a-Reductase, and 5a-Androstane-3,17-dione
